# Emergence of Carbapenem Resistance Due to the Novel Insertion Sequence IS*Pa*8 in *Pseudomonas aeruginosa*

**DOI:** 10.1371/journal.pone.0091299

**Published:** 2014-03-10

**Authors:** Randal C. Fowler, Nancy D. Hanson

**Affiliations:** Creighton University School of Medicine, Department of Medical Microbiology and Immunology, Center for Research in Anti-Infectives and Biotechnology, Omaha, Nebraska, United States of America; The University of Sydney, Australia

## Abstract

Chronic lung infections due to the persistence of *Pseudomonas aeruginosa* in cystic fibrosis patients are typically associated with the emergence of antibiotic resistance. The purpose of this study was to investigate the mechanisms responsible for the emergence of carbapenem resistance when a clinical isolate of *P. aeruginosa* collected from a patient with cystic fibrosis was challenged with meropenem. Nine carbapenem-resistant mutants were selected with subinhibitory concentrations of meropenem from a clinical isolate of *P. aeruginosa* and characterized for carbapenem resistance. Increased carbapenem MICs were associated with the identification of the novel insertion sequence IS*Pa*8 within *oprD* or its promoter region in all the mutants. The position of IS*Pa*8 was different for each of the mutants evaluated. In addition, Southern blot analyses identified multiple copies of IS*Pa*8 within the genomes of the mutants and their parent isolate. These data demonstrate that transposition of IS elements within the *Pseudomonas* genome can influence antibiotic susceptibility. Understanding the selective pressures associated with the emergence of antibiotic resistance is critical for the judicious use of antimicrobial chemotherapy and the successful treatment of bacterial infections.

## Introduction

Carbapenems such as imipenem, meropenem, and doripenem are frequently used as last resort drugs for the treatment of multidrug-resistant *P. aeruginosa* infections. *P. aeruginosa* is the predominate pathogen in chronic respiratory infections in patients with cystic fibrosis. The most formidable characteristic of *P. aeruginosa* is the rapid development of resistance during therapy to multiple antibiotics including the carbapenems, making eradication of this microorganism from the airways of cystic fibrosis patients difficult if not impossible [1], [2]. Although the carbapenems remain a viable therapeutic option, their usage carries an increased risk for developing carbapenem resistance thereby reducing their efficacy [3]–[9].

*P. aeruginosa* can employ a combination of plasmid-encoded and/or chromosomally-encoded mechanisms to evade carbapenem therapy. However, in non-carbapenemase-producing *P. aeruginosa*, meropenem and doripenem resistance is associated with the overexpression of the *mexAB*-*oprM* efflux pump with concomitant loss of the carbapenem-specific porin OprD [10], [11], while imipenem resistance has only been associated with the down-regulation of OprD [10], [12]. Mutations such as nucleotide substitutions, deletions, and insertion sequence (IS) elements within the *oprD* gene or its promoter region can decrease or cause a loss of OprD production, resulting in significant reductions in susceptibility of *P. aeruginosa* to carbapenem antibiotics [13]–[17]. Furthermore, IS21 disruption of the efflux regulator *mexR* has been associated with decreased β-lactam susceptibility due to increased expression of the efflux pump *mexAB*-*oprM* [18]. These data in addition to other studies highlight the various roles IS elements play in promoting the emergence of antibiotic resistance [19].

In this study, we challenged a carbapenem susceptible *P. aeruginosa* isolate collected from a cystic fibrosis patient with subinhibitory concentrations of meropenem and investigated the molecular mechanisms associated with the emergence of carbapenem resistance.

## Methods

### Strains and Culture Conditions

*P. aeruginosa* isolate PA42 was isolated from a sputum culture of a 20 year-old patient with cystic fibrosis from the Pediatrics Pulmonology Cystic Fibrosis Clinic at the University of Nebraska Medical Center. Gram staining revealed that PA42 had a rounded cell morphology compared to the laboratory isolate PAO1 (Figure S1). PA42 was identified as *P. aeruginosa* using the Vitek 2 System. In addition, the sequence data for *oprF* and *oprD* showed 100% and 92% identities to the sequenced strain *P. aeruginosa* PAO1 (GenBank Accession No. AE004091.2).

### Bacterial Susceptibility

PAO1 and PA443, an isolate collected from a cystic fibrosis patient, were susceptible to all anti-pseudomonal agents tested and were used as comparator isolates. Susceptibility of the *P. aeruginosa* isolates to imipenem, meropenem, and doripenem was determined using agar dilution methodology according to the Clinical Laboratory Standards Institute (CLSI) [20]. Quality control strains *E. coli* ATCC 25922, *E. coli* ATCC 35218, and *P. aeruginosa* ATCC 27853 were included in susceptibility testing experiments.

### Generation of Isogenic Mutants

Parent isolate PA42 was challenged with subinhibitory concentrations of meropenem during log-phase growth to generate an isogenic panel of carbapenem-resistant mutants. *P. aeruginosa* cells were harvested by centrifugation, resuspended in sterile saline, and diluted to achieve populations of 10^7^, 10^8^, and 10^9^ cfu/mL. Diluted cultures were inoculated into sets of Mueller-Hinton agar plates containing 0.25 µg/mL or 0.5 µg/mL of meropenem. After 24 h of incubation at 37°C, individual colonies were selected at random from each meropenem plate and putative mutants were evaluated for a ≥4-fold increase in meropenem MIC by agar dilution in accordance with CLSI guidelines [20].

### PCR and Sequencing

Genomic DNA was prepared from all bacterial isolates as previously described [21]. PCR was conducted in a final volume of 50 µL with 2 units of High-Fidelity Platinum® Taq Polymerase (Invitrogen, Carlsbad, CA), 3% DMSO, and primers that flanked either *oprD* or *oprF* (Table S1). The amplification conditions were 95°C for 5 min followed by 25 cycles at 95°C for 30 s, 56°C for 30 s, and 72°C for 3 min. A combination of primers specific for *oprD* and IS*Pa*8 were used in a PCR using a 52°C annealing temperature to determine the location of IS*Pa*8 with respect to the *oprD* gene in the isogenic mutants (Table S1). PCR products were column purified using a Millipore Amicon® Ultra Ultracel® 50 K filter (Millipore, Billerica, MA, USA) and sequenced by ACGT, Inc. (Wheeling, IL).

### Real Time RT-PCR

RNA was isolated from cultures grown to mid-log phase and gene expression was evaluated by real time RT-PCR as previously described [22]. The expression of *mexA* for PA42 and its isogenic mutants was compared to PAO1 and represented as a ratio (fold-change). Expression studies were performed in triplicate with independent bacterial cultures and the relative expression of *mexA* was determined by averaging the results from three independent RNA extractions.

### Outer Membrane Protein Analysis

Outer membrane proteins were isolated and resolved in an 11% SDS–PAGE with a constant current of 25 mA [23]. Proteins were stained with coomassie blue R-250 and photographed using the Molecular Imager® Gel Doc XR+ System with Image Lab Software.

### Pulsed Field Gel Electrophoresis and Southern Hybridization

Pulsed field gel electrophoresis (PFGE) analysis was performed on *P. aeruginosa* genomic DNA digested with *Spe*I as previously described [24]. Southern hybridizations were performed on restricted genomic DNA using methods described by Sambrook and Russell [25]. A 182 bp DIG-labeled probe specific for IS*Pa*8 was synthesized with primers ISPa8F2 and ISPa8R2 (Table S1) using a Roche PCR DIG Probe Synthesis Kit (Roche, Penzberg, Germany) and column purified. The restricted genomic DNA was vacuum blotted to a positively charged nylon membrane and hybridization was carried out with a Roche DIG Nucleic Acid Detection Kit (Roche, Penzberg, Germany) as instructed by the manufacturer.

## Results

### *In vitro* Meropenem Mutant Selection and Susceptibilities

Clinical isolate PA42 was challenged with subinhibitory levels of meropenem. Mutants were isolated from meropenem agar plates and evaluated for carbapenem susceptibilities and mechanisms of carbapenem resistance. The mutational frequency of PA42 challenged with 0.25 µg/mL (⅛ the MIC) of meropenem ranged from 1×10^−7^ to 9×10^−7^, while the mutational frequency was 8×10^−9^ with 0.5 µg/mL (¼ the MIC) of meropenem. In comparison to PA42, the MICs of imipenem and doripenem were 32-fold and 16-fold higher for the selected mutants 711M, 714M, 811M, 812M, 925M, and 927M, while a 4- to 8-fold increase in doripenem MICs and an 8- to 16-fold increase in imipenem MICs were observed for mutants 922M and 924M, respectively. In addition, all the isogenic mutants had a meropenem MIC of 16 µg/mL (Table 1).

**Table 1:** Carbapenem susceptibility and gene expression data for *P. aeruginosa* isolates.

Isolate	Phenotype^a^	Selective Agent^b^	Mutant Frequency	MIC (µg/mL)^c^			Relative *mexA* expression ± SD
				IPM	MEM	DOR	
PAO1	WT	–	–	2	0.5	0.5	1.00
PA443	CF	–	–	2	0.25	0.25	0.85±0.20
PA42	P	–	–	0.5	2	0.25	3.99±1.23
711M	M	0.25	9×10^−7^	16	16	4	3.57±1.10
712M	M	0.25	9×10^−7^	8	16	8	3.62±0.74
714M	M	0.25	9×10^−7^	16	16	4	5.46±1.35
811M	M	0.25	1×10^−7^	16	16	4	4.95±0.85
812M	M	0.25	1×10^−7^	16	16	4	4.14±1.99
922M	M	0.5	8×10^−9^	4	16	1	3.12±0.38
924M	M	0.5	8×10^−9^	8	16	2	4.89±1.48
925M	M	0.5	8×10^−9^	16	16	4	4.02±1.73
927M	M	0.5	8×10^−9^	16	16	4	4.57±1.32

a WT, wild-type isolate; CF, clinical isolate from cystic fibrosis patient; P, parent isolate; M, mutant of PA42.

b Subinhibitory concentration (µg/mL) of meropenem used for mutant selection.

c IPM, imipenem; MEM, meropenem; DOR, doripenem.

### Mechanisms Associated with Carbapenem Resistance

**Figure 1:**
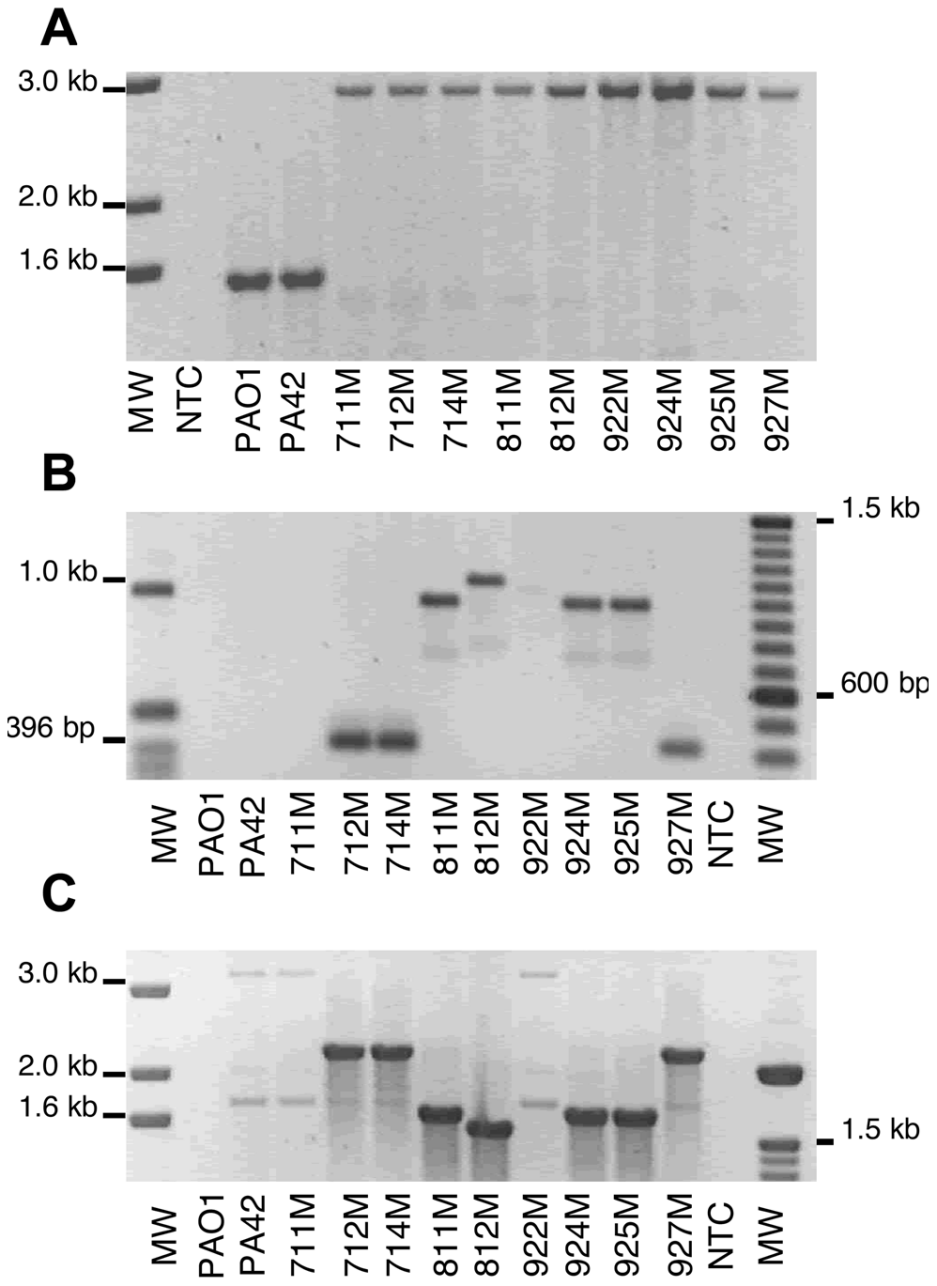
PCR amplification of *oprD* and IS*Pa*8 from carbapenem-resistant mutants of *P. aeruginosa*. (A) Primers that flanked the *oprD* gene were used to PCR-amplify the *oprD* gene giving an expected PCR product size is 1586 bp. Each lane is labeled with its respective DNA ladder (1 kb DNA ladder, Invitrogen), no template control (NTC), or *P. aeruginosa* isolate. (B) PCR amplification of IS*Pa*8 within the *oprD* gene in the nine carbapenem-resistant mutants. Primers ISPa8F1 and OprDRTR3 were used to map the approximate location of the IS*Pa*8 within the *oprD* gene. The smaller PCR products indicate IS*Pa*8 has inserted near the 3′ end of *oprD*, while larger PCR products indicate IS*Pa*8 is inserted near the 5′ end. No PCR product was observed for mutant 711M. (C) Primers OprDRTF2 and ISPa8R2 were used to confirm the location of IS*Pa*8 within the *oprD* gene in the nine isogenic mutants. Non-specific bands were amplified in PA42, mutant 711M, and mutant 922M suggesting that multiple IS*Pa*8 elements may be present within the genome. To confirm that IS*Pa*8 had inserted within the *oprD* gene or its flanking regions in mutants 711M and 922M, PCR products for these mutants shown in (A) were sequenced using primers OprDRTF2 or ISPa8F1.

**Figure 2:**
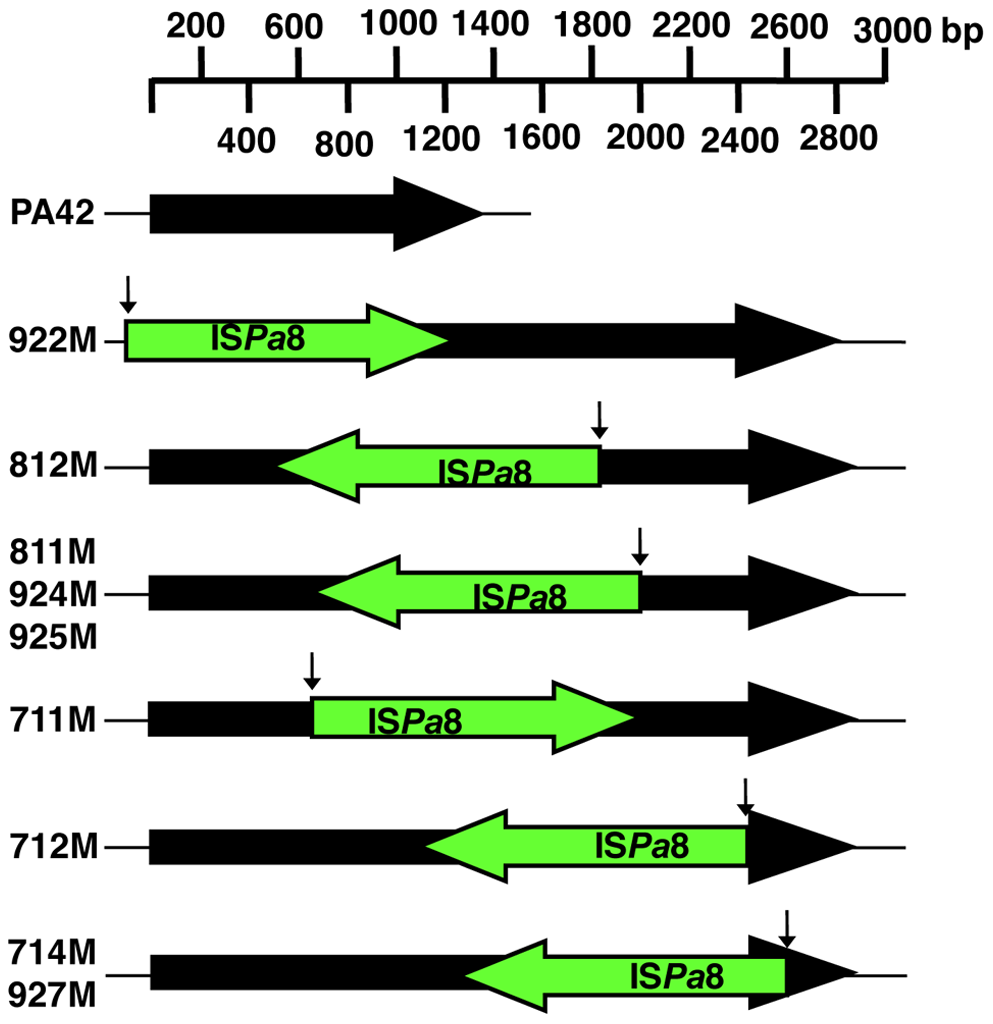
Insertion sites of IS*Pa*8 with respect to the *oprD* gene in PA42 and nine isogenic mutants. The solid black arrows represent the *oprD* gene while the green arrows represent the location and orientation of IS*Pa*8 within *oprD*. The downward arrow indicates the predicted insertion site of IS*Pa*8 within *oprD* as determined by sequence analysis.

Previous studies have shown that a loss of the OprD porin is the most common mechanism associated with reduced susceptibility to the carbapenems in *P. aeruginosa* [2], [13], [14]. It is possible that the elevated carbapenem MICs for the isogenic mutants was associated with mutations within *oprD* that were selected during meropenem exposure (Table 1). To determine whether nucleotide changes within *oprD* had occurred, the *oprD* gene and its flanking regions were amplified by PCR and sequenced. Parent isolate PA42 generated a PCR product of the predicted size (1586 bp) (Figure 1) and sequence analysis of the *oprD* gene from PA42 showed 99% similarity to *oprD* from *P. aeruginosa* LESB58 (GenBank Accession No. FM209186.1) and 92% similarity to *oprD* from *P. aeruginosa* PAO1 (GenBank Accession No. AE004091.2). Surprisingly, the PA42 mutants amplified a much larger PCR product (∼3000 bp) than the PCR product for PA42, which suggested the presence of a large insertion within the *oprD* gene (Figure 1). Sequence analysis of mutant 712M revealed the presence of a 1324 bp IS element located within the *oprD* gene (Accession No. KC710194). The IS element was identified as having 96% similarity to IS*Pa*8 (http://www-IS.biotoul.fr) and was flanked by 19 bp imperfect terminal inverted repeats. An open reading frame within IS*Pa*8 was found to encode a 364 amino acid protein having 84% identity to a putative transposase in *Pseudoxanthomonas suwonensis* (GenBank Accession No. CP002446.1). PCR analysis using a combination of IS*Pa*8 and *oprD*-specific primer sets listed in Table S1 revealed that IS*Pa*8 had also inserted within *oprD* or its promoter region in the other isogenic mutants (Figure 1). To identify the insertion site and confirm the IS element was IS*Pa*8, a single sequence reaction was performed using primers listed in Table S1. PCR and sequence analysis determined that the disruption of *oprD* in eight mutants was due to IS*Pa*8 insertion, while the insertion of IS*Pa*8 was found 57 bp upstream of the translational start codon of *oprD* in mutant 922M. Although inactivation of the *oprD* gene in the isogenic mutants was due to IS*Pa*8, the position and orientation of this IS element was different with respect to *oprD* (Figure 2).

Meropenem and doripenem resistance can emerge through a combination of down-regulation or loss of OprD in the outer membrane and an up-regulation of MexAB-OprM in non-metallo-β-lactamase producing *P. aeruginosa* isolates [2], [11]. To account for the higher MICs of meropenem and doripenem in the isogenic mutants, we compared the relative transcript levels of *mexA* in the isogenic mutants, their parent PA42, and a fully susceptible cystic fibrosis isolate PA443 to *mexA* transcript levels in PAO1. *mexA* expression was 4-fold higher in PA42 than PAO1 and PA443, indicating that *mexA* expression was already elevated above “wild-type” levels observed for PAO1 and PA443. However, *mexA* transcript levels were similar among the isogenic mutants and their parent, PA42 suggesting that *mexA* expression did not change upon meropenem selection (Table 1).

### Outer Membrane Profiles of PA42 and Isogenic Mutants

**Figure 3:**
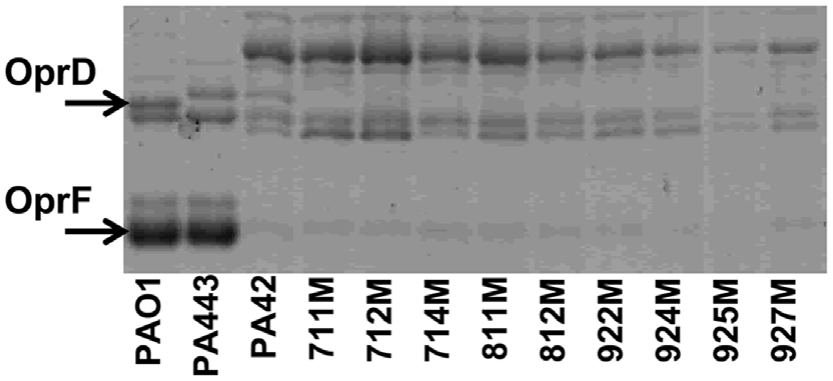
Outer membrane analysis of *P. aeruginosa* PA42 and nine isogenic mutants. Outer membrane profiles were analyzed for the presence of the porin, OprD. PAO1 and a fully susceptible cystic fibrosis isolate, PA443, were included as positive controls. The locations of OprD and OprF proteins are indicated.

**Figure 4:**
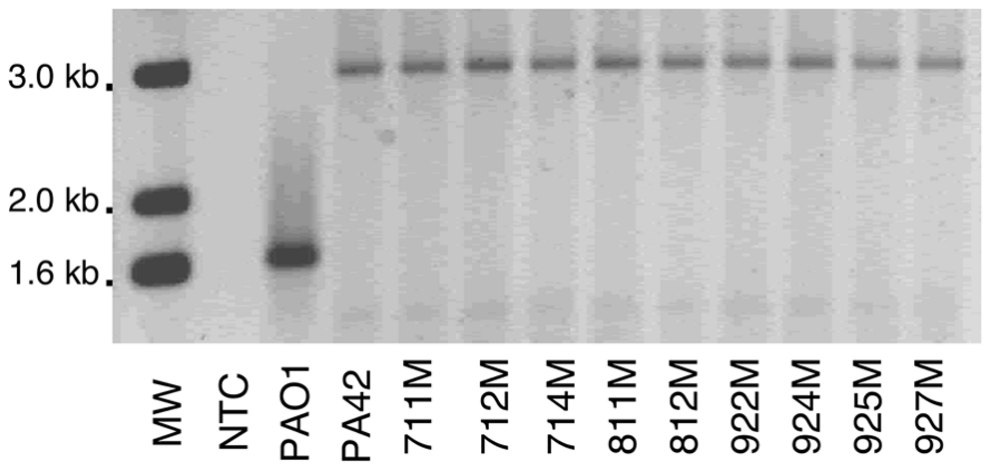
PCR amplification of the *oprF* gene in PAO1, PA42, and nine isogenic mutants. The expected PCR product size is 1,672(1 kb DNA ladder, Invitrogen), no template control (NTC), or *P. aeruginosa* isolates.

The inactivation of *oprD* by IS*Pa*8 suggested that the OprD protein was no longer produced. Therefore, the presence of OprD in the outer membrane was analyzed in the isogenic mutants. The outer membrane profiles for PA42 and its mutants showed that OprD was produced in PA42 but not in the isogenic mutants (Figure 3). The loss of OprD correlated with the insertional inactivation of the *oprD* gene by IS*Pa*8 and the reduced carbapenem susceptibility in the isogenic mutants. In addition to the loss of OprD in the mutants, parent isolate PA42 and the isogenic mutants did not produce the major outer membrane protein OprF (Figure 3). To investigate the potential source of IS*Pa*8 on the chromosome of PA42, we sought to identify whether the loss of OprF was also the result of IS*Pa*8 insertion within the *oprF* gene. To test this hypothesis, the *oprF* gene and its flanking regions were PCR amplified and sequenced. PCR amplification of *oprF* from PA42 generated a much larger product (∼3000 bp) than the predicted 1672 bp product from PAO1 (Figure 4). Sequence analysis of *oprF* for PA42 identified the insertion of IS*Pa*8 31 nucleotides upstream of the translational start codon of *oprF* (Accession No. KC710195). The disruption of the *oprF* promoter region by IS*Pa*8 in parent isolate PA42 correlated with a 50-fold decrease in *oprF* expression compared to *oprF* expression in PAO1 (data not shown). Additionally, the rounded cell shape observed for PA42 could be linked to the loss of OprF as previously reported [26]–[28]. Subsequently, amplification of the *oprF* gene in the isogenic mutants resulted in PCR products that were ∼1,400 bp larger than expected (Figure 4). OprF was absent or produced at low levels in the outer membranes of PA42 and its mutants (Figure 3). Taken together, these data indicated that IS*Pa*8 remained upstream of *oprF* during meropenem exposure and was responsible for the lack of OprF production in PA42 and the isogenic mutants.

### PFGE Patterns and IS*Pa*8 Copy Number

**Figure 5:**
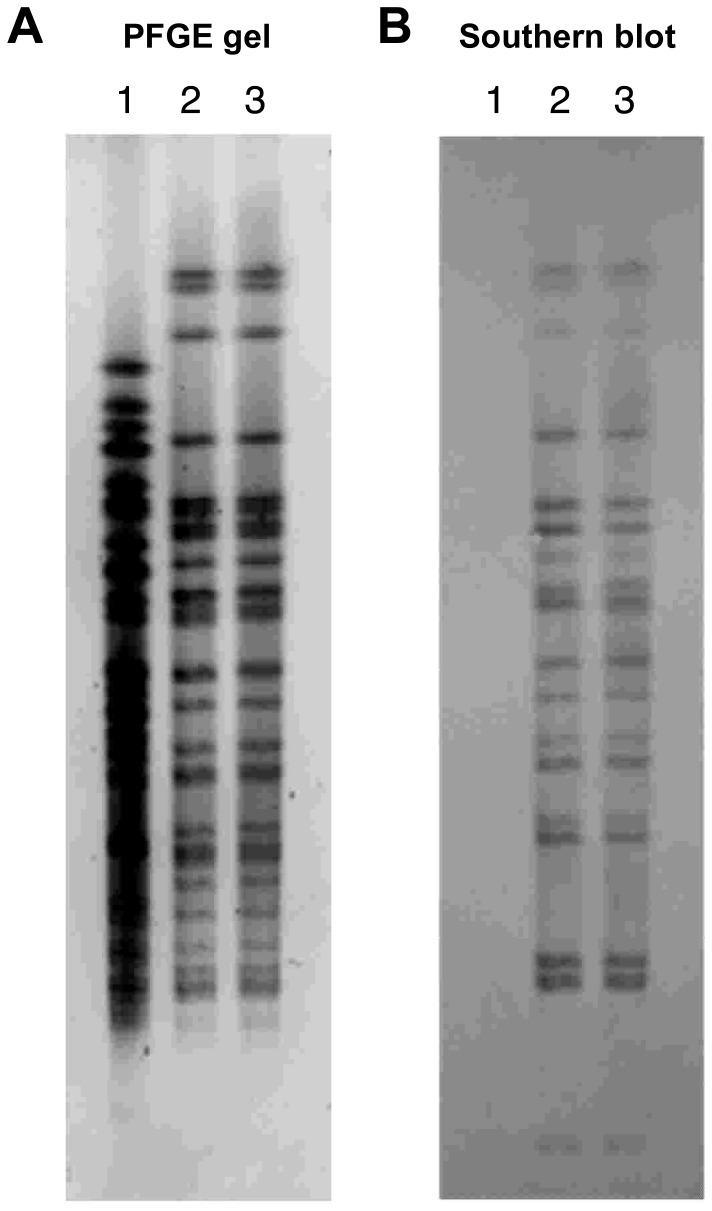
Pulsed Field Gel Electrophoresis and Southern blot analyses using IS*Pa*8-specific probe. (A) PFGE gel of *Spe*I chromosomal digests of wild-type strain PAO1, parent isolate PA42, and mutant 812M visualized using SYBR gold. (B) Southern blot of the gel depicted in Figure 5(A) using an IS*Pa*8-specific probe. Lane 1, PAO1 (negative control); lane 2, parent isolate PA42; lane 3, mutant 812M.

To determine the copy number of IS*Pa*8 in PA42 and its mutants, PFGE and Southern blot experiments were performed. As anticipated, PA42 and its mutants were highly related by PFGE as indicated by their indistinguishable PFGE patterns (Figure 5, Figure S2). Surprisingly, Southern blot analysis revealed multiple hybridization signals for IS*Pa*8 in PA42 and mutant 812M demonstrating that multiple copies of IS*Pa*8 exist in their genomes (Figure 5). Note that in Figure 5 the DIG-probe was highly specific for IS*Pa*8 as no hybridization signal was observed in the IS*Pa8*-negative control PAO1. Multiple copies of IS*Pa*8 were also observed in the genomes of the other mutants (Figure S2). However, we could not differentiate the number of hybridization signals between PA42 and each mutant due to the high copy number (>20 copies) of the IS*Pa*8 within their genomes as illustrated in the analysis of PA42 and mutant 812M (Figure 5).

## Discussion

Carbapenem resistance poses a major therapeutic challenge for the treatment of *P. aeruginosa* infections, particularly in patients with cystic fibrosis. Our current understanding of the mechanisms responsible for carbapenem resistance consists of an interplay between the over-expression of *mexAB*-*oprM* and the down-regulation of the porin, OprD [2], [10], [11]. The most common mechanism of imipenem resistance is the absence of OprD production which can result from mutations, insertions, and/or deletions in the *oprD* gene as well as the down-regulation of *oprD* transcription [13]–[17]. In this study, the insertion of a novel IS element, identified as IS*Pa*8, within the *oprD* gene was associated with the emergence of carbapenem resistance in *P. aeruginosa* mutants selected with subinhibitory levels of meropenem. These findings are in agreement with other investigations where IS element insertion has been linked to a decrease in carbapenem susceptibility and loss of OprD production. These observations indicate that the *oprD* gene and/or its promoter region may serve as a hot spot in the genome for IS element insertion [15]–[17], [29].

The loss of OprD in the outer membrane has been shown to decrease the susceptibility of *P. aeruginosa* by 4- to 16-fold for imipenem, compared to 4- to 32-fold for meropenem and 8- to 32-fold for doripenem [11], [30], while up-regulation of *mexAB-oprM* expression increased the MICs of meropenem and doripenem from 0.12–0.5 µg/mL to 4 µg/mL [11]. However, a concomitant loss of OprD and up-regulation of this efflux pump can increase the MIC of meropenem above “wild-type” levels (∼16 µg/mL) [10]. In this study, meropenem resistance (16 µg/mL) among the isogenic mutants resulted from increased *mexA* transcript levels that preceded meropenem exposure in addition to a loss of OprD through insertional disruption by IS*Pa*8 allowing for the selection of the meropenem mutants. Interestingly, the parent isolate from which carbapenem-resistant mutants were selected showed a 4-fold increase in *mexA* expression compared *mexA* expression in PAO1 which correlated with the MIC of meropenem (2 µg/mL). However, meropenem selection did not modify the expression of *mexA* in the isogenic mutants compared to the parent isolate PA42, suggesting that *mexA* expression may have reached an expression threshold sufficient to achieve high meropenem MICs (Table 1). However, as previous described [10], it is the combination of increased *mexA* expression and loss of OprD as displayed in these isogenic mutants that resulted in the MICs of doripenem and meropenem to increase above the resistance breakpoint, while the loss of OprD alone was responsible for the reduction in susceptibility to imipenem in these mutants. These data demonstrate that OprD-mediated resistance is not always the first step toward carbapenem resistance as has been previously suggested [10], [11]. Taken together, these data further support that a combination of chromosomally-encoded resistance mechanisms are necessary to achieve meropenem and/or doripenem MICs above the resistance breakpoint.

Transposable elements such as IS elements can promote the adaptation and survival of Gram-negative bacteria under different environmental niches through the disruption of genes and genomic rearrangements. This form of adaptation has been well documented in clinical isolates of *P. aeruginosa* that have developed resistance to β-lactam antibiotics [15]–[19]. Moreover, IS elements modulate genomic plasticity and participate in the evolution of *P. aeruginosa* isolates within the lung environment of patients with cystic fibrosis through genetic rearrangements [31]. The presence of multiple copies of IS*Pa*8 in the host chromosome of PA42 suggests that it may have played an important role in the evolution of this clinical isolate. Recently, Doumith *et al* suggested that bacterial chromosomes may contain multiple copies of IS elements and their insertion into porin genes may be selected under antibiotic pressure [32]. To our knowledge, our study is the first to provide support that when a selective pressure is applied to a bacteria with multiple copies of an IS element, antibiotic-resistant mutants with insertional disruptions in porin genes will be selected for within the bacterial population. Interestingly, multiple insertion events of IS*Pa*8 occurred within the *oprD* gene or promoter region among the meropenem selected mutants, which may be a reflection of the host genome containing several copies of the IS*Pa*8 element (Figure 2). These data suggest that *P. aeruginosa* isolates with a high copy number of IS elements within its genome could lead to the emergence of antibiotic resistance. This observation could have serious implications in clinical practice where carbapenem monotherapy is used as the primary treatment of *P. aeruginosa* infections. Ruiz-Martinez *el al* reported a similar finding where imipenem monotherapy was used to treat a fully susceptible *P. aeruginosa* infection in a patient, which resulted in the selection of an imipenem-resistant variant due to the inactivation of the *oprD* gene by IS*Pa*133 [29]. Taken together, these data indicate that the selective pressure exerted by a carbapenem can select mutants from *P. aeruginosa* isolates that contain multiple copies of IS element in the genome thereby allowing for the rapid development carbapenem resistance.

This study serves as an important reminder that monotherapy, particularly with a carbapenem, may not always be appropriate for treating *P. aeruginosa* infections as it can readily develop resistance to antibiotics. Although IS elements have been shown to influence the genetic and phenotypic characteristics of bacteria as described in this study, little is known about what stimulates their transposition. Therefore, future studies looking to identify factors that influence the mobilization and insertion of IS elements could contribute significantly to our current knowledge of mechanisms involved in the emergence of antibiotic resistance and the evolution of *P. aeruginosa*.

## Supporting Information

Figure S1 **Macroscopic and microscopic analysis of** ***P. aeruginosa*** **isolates PAO1 and PA42.** Macroscopic analysis of PAO1 grown on blood agar shows a non-mucoid phenotype while PA42 was mucoid when grown on blood agar. Gram-staining revealed characteristic Gram-negative rods for PAO1 and unique Gram-negative cocci for PA42 at 40X and 100X objectives.
(PDF)

Figure S2 **Pulsed Field Gel Electrophoresis and Southern blot analyses using IS** ***Pa*** **8-specific probe.** (A) PFGE gel of *Spe*I chromosomal digests of wild-type strain PAO1, parent isolate PA42, and eight carbapenem-selected mutants visualized using SYBR gold. Lane 1, size standard *Staphylococcus aureus* NCTC 8325; lane 2, negative control PAO1; lane 3, parent isolate PA42; lane 4, mutant 711M; lane 5, mutant 712M; lane 6, mutant 714M; lane 7, mutant 811M; lane 8, mutant 922M; lane 9, mutant 924M; lane 10, mutant 925M; lane 11, mutant 927M. (B) Southern blot of the gel depicted in Figure S2(A) using an IS*Pa*8-specific probe. Lane 1, size standard *Staphylococcus aureus* NCTC 8325; lane 2, negative control PAO1; lane 3, parent isolate PA42; lane 4, mutant 711M; lane 5, mutant 712M; lane 6, mutant 714M; lane 7, mutant 811M; lane 8, mutant 922M; lane 9, mutant 924M; lane 10, mutant 925M; lane 11, mutant 927M.
(PDF)

Table S1 Primers used in this study.
(DOCX)
