# Supplementary figures and images for: Emergence of Carbapenem Resistance Due to the Novel Insertion Sequence ISPa8 in Pseudomonas aeruginosa
Source: PLoS One. 2014 Mar 10;9(3):e91299. doi: 10.1371/journal.pone.0091299 (PMC3948848; doi:10.1371/journal.pone.0091299)

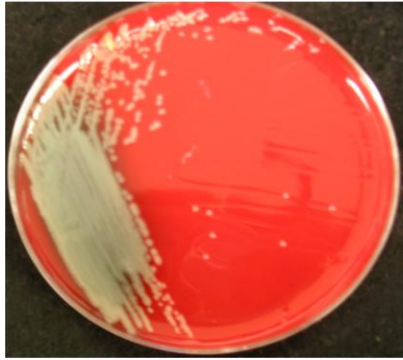

PAO1

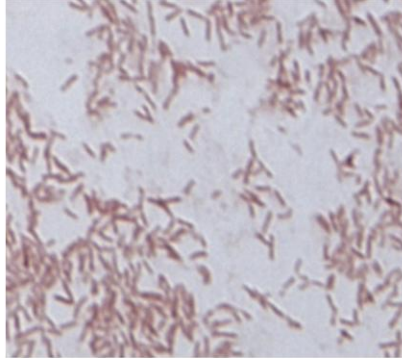

(40X)

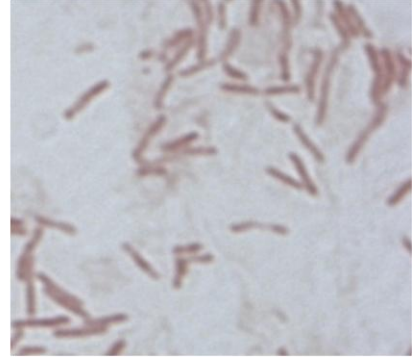

(100X)

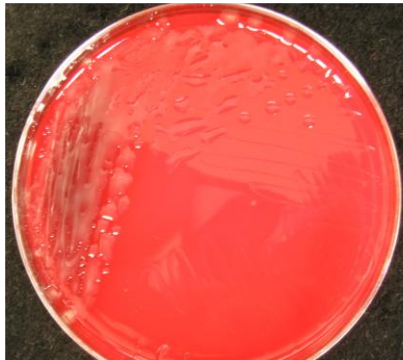

PA42

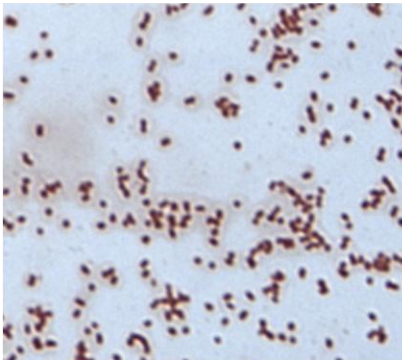

(40X)

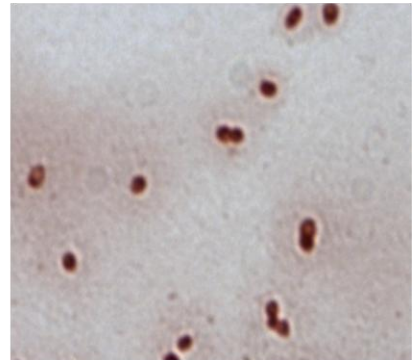

(100X)

Supplement: Figure S1 — Macroscopic and microscopic analysis of P. aeruginosa isolates PAO1 and PA42. Macroscopic analysis of PAO1 grown on blood agar shows a non-mucoid phenotype while PA42 was mucoid when grown on blood agar. Gram-staining revealed characteristic Gram-negative rods for PAO1 and unique Gram-negative cocci for PA42 at 40X and 100X objectives. (PDF) [file pone.0091299.s001.pdf]

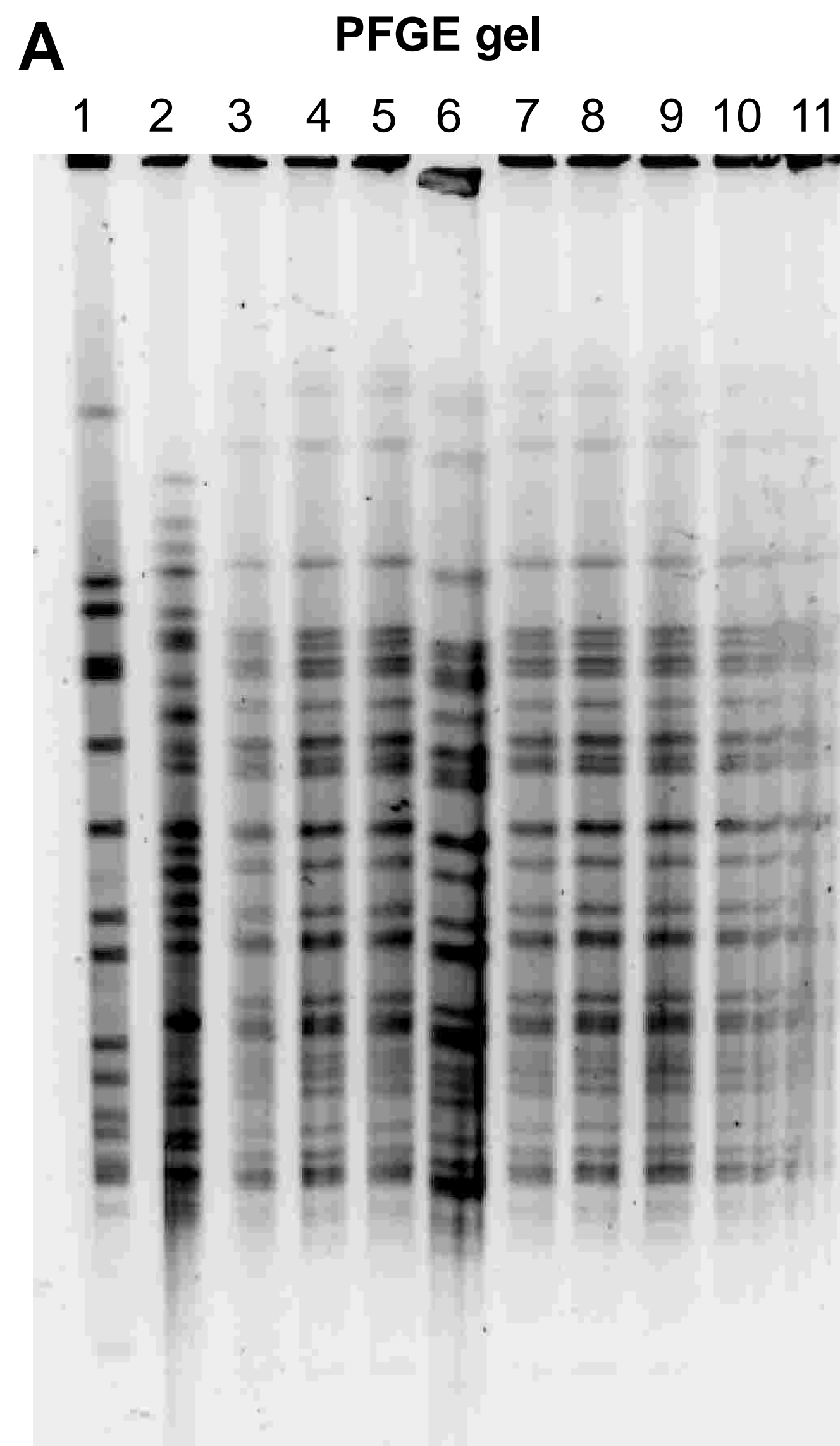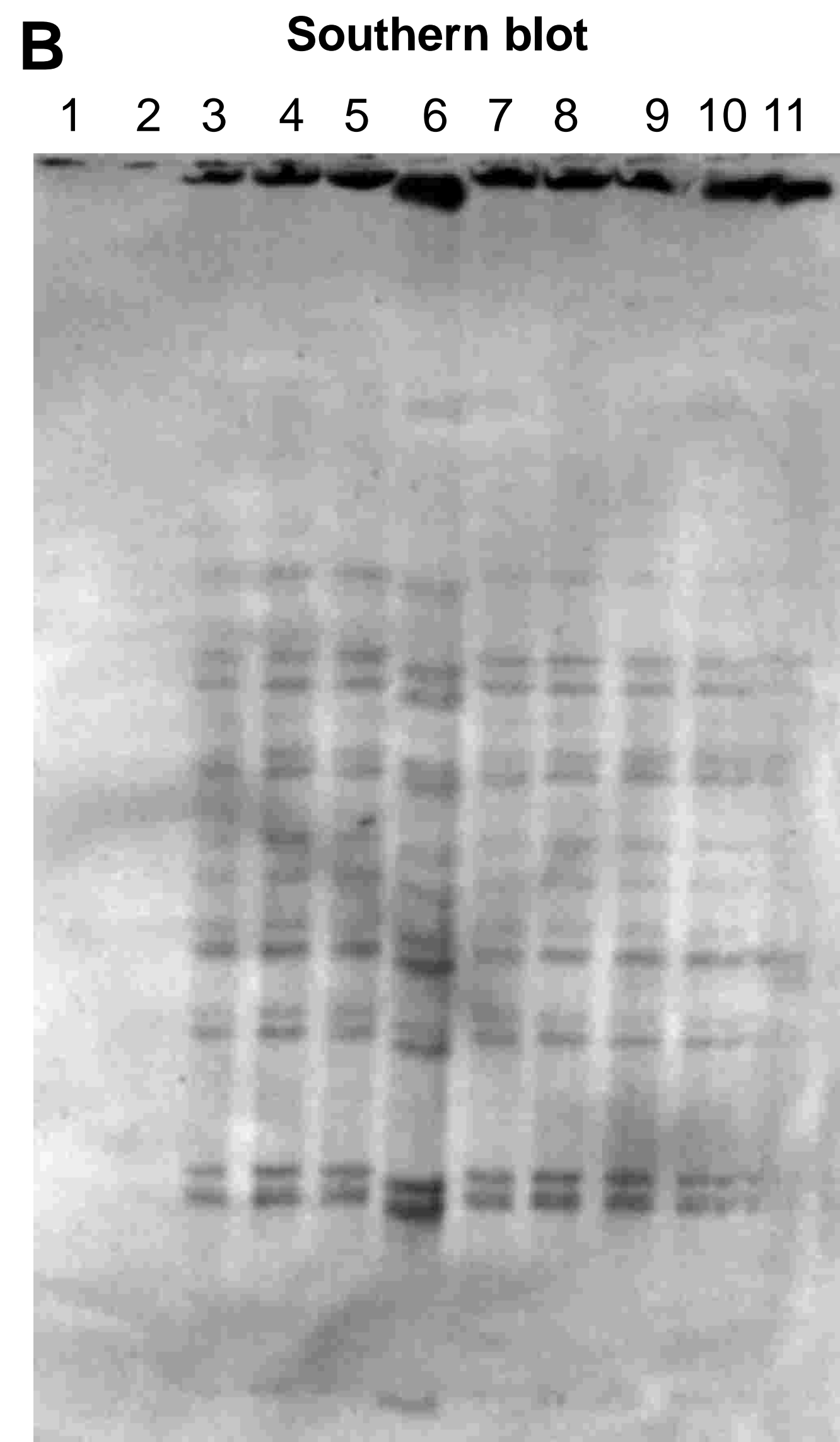

Supplement: Figure S2 — Pulsed Field Gel Electrophoresis and Southern blot analyses using IS Pa 8-specific probe. (A) PFGE gel of SpeI chromosomal digests of wild-type strain PAO1, parent isolate PA42, and eight carbapenem-selected mutants visualized using SYBR gold. Lane 1, size standard Staphylococcus aureus NCTC 8325; lane 2, negative control PAO1; lane 3, parent isolate PA42; lane 4, mutant 711M; lane 5, mutant 712M; lane 6, mutant 714M; lane 7, mutant 811M; lane 8, mutant 922M; lane 9, mutant 924M; lane 10, mutant 925M; lane 11, mutant 927M. (B) Southern blot of the gel depicted in Figure S2(A) using an ISPa8-specific probe. Lane 1, size standard Staphylococcus aureus NCTC 8325; lane 2, negative control PAO1; lane 3, parent isolate PA42; lane 4, mutant 711M; lane 5, mutant 712M; lane 6, mutant 714M; lane 7, mutant 811M; lane 8, mutant 922M; lane 9, mutant 924M; lane 10, mutant 925M; lane 11, mutant 927M. (PDF) [file pone.0091299.s002.pdf]
