# Supplementary material for: Emergence of Carbapenem Resistance Due to the Novel Insertion Sequence ISPa8 in Pseudomonas aeruginosa
Source: PLoS One. 2014 Mar 10;9(3):e91299. doi: 10.1371/journal.pone.0091299 (PMC3948848; doi:10.1371/journal.pone.0091299)
Supplement: Table S1 — Primers used in this study. (DOCX) [file pone.0091299.s003.docx]

Table S1. Primers used in this study.

| **Primer** | **Target** | **Sequence (5’- 3’)** | **Application** | **Reference** |
| --- | --- | --- | --- | --- |
| OprDSEQF1 | *oprD* | CTACGCAGATGCGACATGC | PCR/Sequencing | [15] |
| OprDSEQR1 | *oprD* | CCTTTATAGGCGCGTTGCC | PCR/Sequencing | [15] |
| OprDRTF2 | *oprD* | GAAAGTGATGAAGTGGAGCG | PCR/Sequencing | This study |
| OprDRTF3 | *oprD* | GAAGCCAAGTACGTGGTCCAG | PCR/Sequencing | [32] |
| OprDRTR3 | *oprD* | CAGGATCGACAGCGGATAGTC | PCR/Sequencing | [32] |
| ISPa8F1 | IS*Pa*8 | CGGAACAGCAGGTTGTAGC | PCR/Sequencing | This study |
| ISPa8F2 | IS*Pa*8 | CAAGGACGATACGCCAGACG | Southern blot | This study |
| ISPa8R2 | IS*Pa*8 | GGTGTTCCATCAGCACATGC | Southern blot | This study |
| FF1-oprF | *oprF* | CGGATTGGATCGTTGGCTCG | PCR/Sequencing | This study |
| FR1-oprF | *oprF* | CACGCTGGTGGTCTACATGG | PCR/Sequencing | This study |
| MexARTF | *mexA* | CAAGCAGAAGGCCATCCTC | Expression | [32] |
| MexARTR2 | *mexA* | CGGTAATGATCTTGTCGCCG | Expression | [32] |
| RpsLF1 | *rpsL* | GCAACTATCAACCAGCTGGTG | Expression | [32] |
| RpsLR1 | *rpsL* | GCTGTGCTCTTGCAGGTTGTG | Expression | [32] |
